# Sporozoite egress from *Plasmodium* oocysts requires a trimeric NF-Y–like complex

**DOI:** 10.1038/s42003-026-10147-6

**Published:** 2026-04-27

**Authors:** Claude Marie François Preira, Elena Deligianni, Maria Andreadaki, Sofia Tzagkaraki, Emmanouil Kokkas, Aneta Kołakowska, Renate Gessmann, Chiara Bertaso, Simona Masiero, Rosaria Russo, Marco Nardini, Inga Siden-Kiamos, Louise J. Gourlay, Chiara Currà

**Affiliations:** 1 Institute of Molecular Biology and Biotechnology, FORTH, Heraklion, Greece; 2 Department of Biology, University of Crete, Heraklion, Greece; 3 Department of Biosciences, University of Milano, Milano, Italy; 4 Department of Pathophysiology and Transplantation, University of Milano, Milano, Italy; 5 Department of Infectious Diseases, Italian National Health Institute, Rome, Italy

**Keywords:** Parasite biology, Parasite genetics

## Abstract

The oocyst, the sporogonic stage of the malaria parasite located on the mosquito gut, is protected by a capsule, or cyst wall, which surrounds the plasma membrane. The capsule opens to release the infectious sporozoites. Previously, we identified two Oocyst Rupture Proteins (ORP1-2) that are essential for capsule excystation and sporozoite egress. Both ORPs contain a Histone-Fold Domain similar to the NF-YB and -YC subunits of the trimeric human transcription factor NF-Y. Here we identify a *Plasmodium* protein, named ORP3, as a third subunit of the ORP complex. Although ORP3 is largely unrelated to the NF-YA subunit responsible for trimerization with DNA-binding specificity of NF-Y, it retains the well conserved NF-YA helix A1, responsible for trimerization with the NF-YB/C dimer. A detailed phenotypic analysis of an *orp3(-)* mutant showed a defect in oocyst opening, as observed for the for *orp1(-)* and *orp2(-)* mutants. ORP3 localizes to the periphery of the oocyst, consistent with its position at the capsule. The functional importance of the conserved helix A1 of ORP3 was confirmed, as deletion of this helix abolishes oocyst excystation. The formation of a trimeric complex between a construct containing the helix A1 with the ORP1/2 dimer was further confirmed in vitro using a Yeast-3-hybrid approach. Finally, we confirmed the motility of *orp(-)* sporozoites, suggesting that the block in parasite transmission following injection into naïve mouse is likely due to a failure in developing into exoerythrocytic forms. Our data strengthen the hypothesis that *Plasmodium* has re-purposed the NF-Y complex fold and assembly for the unique biological function of promoting oocyst excystation and sporozoite release.

## Abstract (web summary)

ORP3 is a newly identified *Plasmodium* protein forming a trimeric complex with ORP1/2, essential for oocyst opening and sporozoite release. Its conserved helix A1 enables complex formation, and its loss blocks parasite transmission.

## Introduction

The infectious stages of the malaria parasite, the sporozoite, develop in the oocyst that resides in the mosquito midgut. The oocyst is the sporogonic stage of the parasite, and within a single oocyst, more than a thousand sporozoites are formed approximately 14 days after the uptake of a blood meal. About 13 mitotic divisions take place in a syncytium, concomitant with organelle replication, leading to sporozoite formation inside the so-called sporoblasts. The oocyst is protected by a capsule or cyst wall of mostly unknown composition, which externally surrounds the plasma membrane. During the final stages of development, the plasma membrane separates the cytoplasm, forming the sporoblasts, until the oocyst cell membrane completely disappears. Once the sporozoites have matured, the capsule weakens through the formation of small holes, releasing the sporozoites into the hemocoel before transfer to the salivary glands where they are transmitted to a new vertebrate host during blood feeding^1–3^.

Sporozoite egress is essential for completion of the malaria parasite’s life cycle in the mosquito and thus, understanding this process may reveal new targets for transmission-blocking strategies. Despite this, little is known on the subject, partly because the oocyst is difficult to study as it is located beneath the basement membrane, in close contact with the midgut epithelial cells. In addition, the oocyst capsule composition is poorly characterized and only three parasite-derived capsule proteins have been described to date: Cap380, Cap93, and OSCP, along with two putative capsule-associated proteins named ORP1 and ORP2 that we previously described^4–7^. In addition, thrombospondin-related protein 1 (TRP1) has been shown to mediate sporozoite egress and confer motility to sporozoites before capsule rupture^1^. Proteins of mosquito origin have also been suggested to be incorporated into the capsule^8^. With respect to capsule composition, excystation is even less well understood. Ultrastructural analyses suggest that a single pore forms in the capsule through which the now motile sporozoites egress^9^. Another study, employing a fluorescent strain, showed that upon sporogony completion, the capsule is weakened through the formation of holes, leading to sporozoite release^2^. A few proteins have been implicated in this process, although the molecular mechanisms are unknown and mutational analyses suggest that rupture depends on coordinated interplay between oocyst and sporozoite proteins, highlighting the essential role of sporozoite motility for capsule rupture^10^. The circumsporozoite protein (CSP), a component of the oocyst plasma membrane, is essential for successful sporozoite development and represents a major sporozoite surface protein^11^. CSP contains several well-characterized domains; in a mutant lacking the so-called central repeat region, sporozoites form but fail to egress^12^. Whether this defect is due to a role of CSP in the oocyst or in the sporozoite, however, is unclear. A deletion mutant lacking the cysteine protease ECP1 develops into mature sporozoites, which although motile, remain trapped inside the oocyst^13^. This protein has been identified in sporozoites, but its presence in oocysts is unknown^13^. Importantly, two Oocyst Rupture Proteins (ORP1-2) are essential for sporozoite release and deletion mutants lacking either ORP form sporozoites, but they cannot egress from the oocyst^7,14^.

Both ORPs contain a histone-fold domain (HFD), a short (about 150 amino acids) α-helical domain, commonly found in histones and transcription factors. The HFDs of ORP1 and ORP2 are similar to those of the NF-YB and NF-YC subunits, respectively, of the trimeric human transcription factor NF-Y, specific for binding to the CCAAT-box^15^. High-resolution structural data have revealed that NF-YB and NF-YC form a highly stable HFD dimer that binds DNA non-specifically, similarly to core histones, while CCAAT-box specificity is provided by the non-HFD NF-YA subunit^16^. Mutant parasites expressing either ORP1 or ORP2, devoid of their HFDs, exhibit blocked oocyst rupture^7,14^. Homology modeling and = crystallographic analysis (PDB: 9RIF) of the two ORP HFDs revealed that they assemble into a dimer that is structurally similar to NF-YB/YC^7,17^.

The 3D structure of the NF-Y trimer (PDB: 4AWL) revealed two α-helices, A1 and A2, of the third subunit, NF-YA^17^. Helix A1 mediates trimerization with the NF-YB/YC dimer, whereas helix A2, together with a Gly-rich GGRFF motif, is responsible for DNA recognition and specific binding to the CCAAT-box found in promoters regulated by NF-Y^15^.

Here, we identify a third subunit of the putative trimeric ORP complex in *Plasmodium berghei*, named Oocyst Rupture Protein 3 (ORP3). Our data indicate that ORP3 interacts with the ORP1/2 dimer through a region similar to helix A1 of NF-YA in the NF-Y trimer, and that ORP trimerization is essential to promote oocyst opening and sporozoite release.

## Results

### A *Plasmodium* homolog of NF-YA is essential for sporozoite release from mature oocysts

**Fig. 1:**
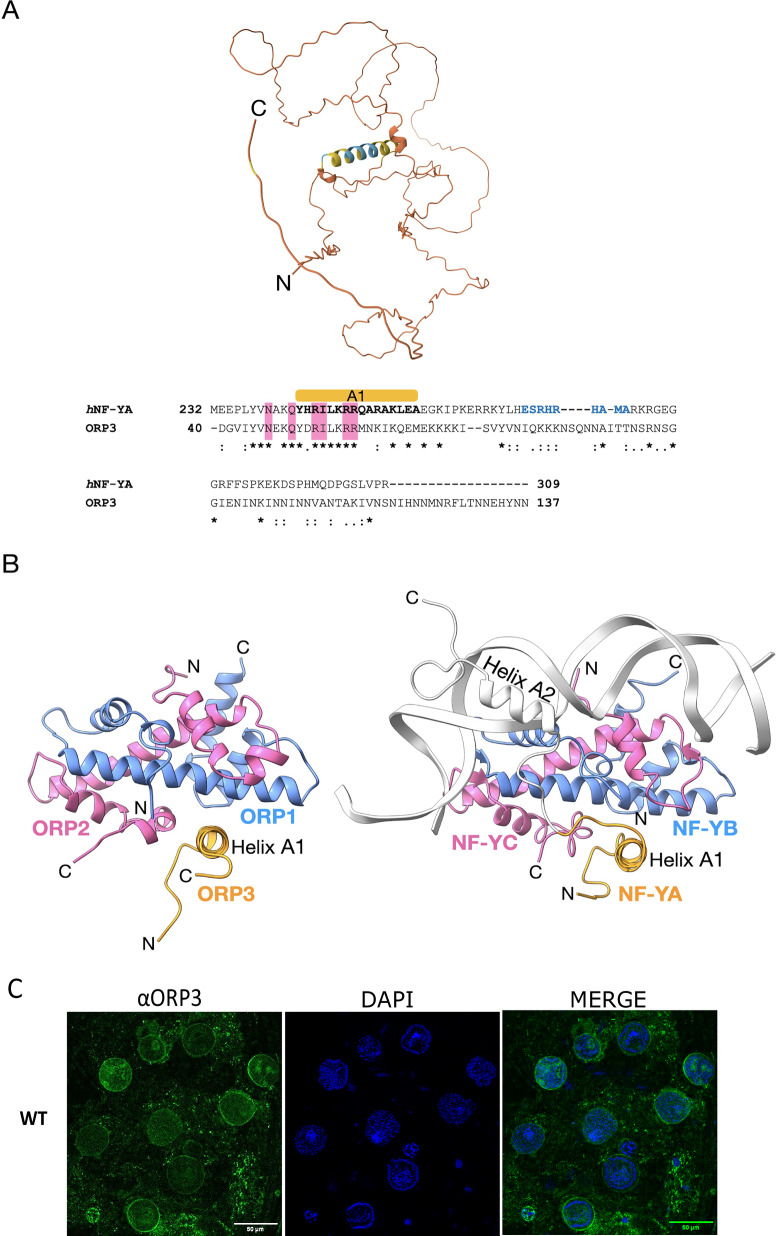
ORP3, a NF-YA subunit homolog is expressed at the oocyst periphery. **A** Upper panel: The 3D structure of full-length ORP3 predicted using Alphafold (Accession code A0A509AM99), shown in ribbons and colored according to the Alphafold plDDT confidence score, with very low confidence regions in orange (plDDT <50), low confidence regions in yellow (plDDT >50), high confidence regions in turquoise (plDDT >70) and very high confidence regions (not present in ORP3) in blue (plDDT >90)^17^. The N- and C-termini are labeled. Lower panel: Sequence alignment of the amino acid residues of the human NFY-A subunit present in PDB:4AWL^15^ and the N-terminal ORP3 construct (PBANKA_0928400; residues 40–137) used in yeast-3-hybrid assays was carried out using ClustalOmega^37^. The aligned sequences share an identity of 30.7%, whereas sequence identity at the level of α-helix A1 in NF-YA (residues 243–258) increases to 56.25%. The α-helix A1 of NF-YA is shown in yellow, and its corresponding residues are shown in bold font. NF-YA residues belonging to the DNA-binding helix (α-helix A2) that is interrupted in ORP3, are shown in blue bold font. Residues described in ref. ^15^ that are key for oligomerization between NF-YA and NF-YB/YC are highlighted in pink shading together with the conserved residues in ORP3; **B**. Left panel: The ORP trimer, predicted using the AlphaFold Server^17^. ORP1 (blue), ORP2 (pink), and ORP3 (orange) are shown in ribbons. Right panel: the NF-Y trimer (PDB: 4AWL^15^ with A, B and C subunits in orange, blue, and pink ribbons, respectively. Bound DNA and extra secondary structure elements present in NF-YA, including helix A2 involved in DNA binding are shown in white ribbons. The N- and C-termini of all chains are shown. **C** WT oocyst probed with a specific ORP3 serum (green) while nuclei are stained with DAPI (blue). Panels A and B were generated using ChimeraX version 1.9^38^.

Reasoning that, in addition to homologs of the B and C subunits of the NF-Y complex, a subunit A might exist in *Plasmodium*, we carried out sequence-based similarity searches and identified a putative homolog. The protein, here named ORP3 (accession code PBANKA_0928400), is annotated as a protein of unknown function in the *Plasmodium* database PLASMODB^18^. ORP3 consists of 358 amino acids and contains, as the only annotated domain, the NFYA_HAP2_2 domain (PROSITE accession code PS51152). This short region (residues 47–71) corresponds to helix A1 in NF-YA that mediates trimerization with the NF-YB/YC dimer. No other domains or motifs are present, and AlphaFold^19^ predicts the protein to be largely, intrinsically disordered (upper panel Fig. 1A) except for NFYA_HAP2_2 domain region, which adopts an α-helix that similar to helix A1 (lower panel Fig. 1A). Outside this region, no similarity exists with NF-YA, nor with any other protein outside the *Plasmodium* genus (Protein BLAST of non-redundant database at NCBI https://blast.ncbi.nlm.nih.gov/Blast.cgi?PAGE=Proteins). A comparison of helix A1 in NF-YA (residues 243–258) with the corresponding residues in ORP3 (residues 50–65) shows a sequence identity of 56%. In addition to helix A1, conserved residues are also present in flanking regions (lower panel Fig. 1A). Sequence analyses revealed that the second α-helix of NF-YA, helix A2, which is required for DNA binding, is not present in ORP3 (lower panel Fig. 1A), in line with its non-nuclear localization. A trimer complex between the HFD dimer of ORP1 (residues 774–860) and ORP2 (residues 5–111) with residues 40–72 of ORP3, including helix A1, was predicted using the AlphaFold server^19^, resulting in a very high confidence model (plDDT > 90) (Fig. 1B and Supplementary Fig. 1). Comparison of the structural organization of the ORP trimer with the NFY trimer, reveals a similar mode of oligomerization (Fig. 1B). Sequence alignment of the putative interface region between ORP3 and the ORP1-ORP2 dimer interface, with that of NF-YA with the NF-YB/YC dimer, illustrates that the amino acids involved in the most relevant polar and hydrophobic interactions required for trimer stability in NF-Y are 100% conserved in ORP3 (Fig. 1A; 16).

**Fig. 2:**
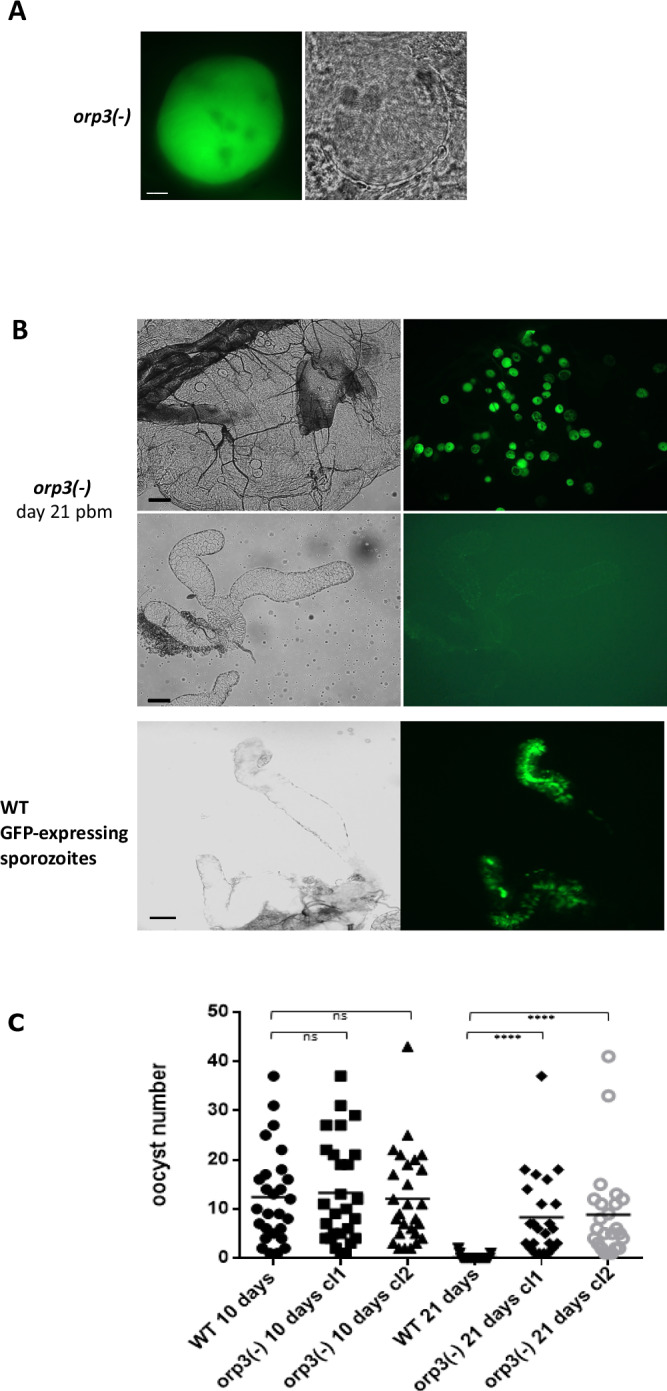
Phenotypic analysis of the *orp3(−)* mutant. **A** A mature *orp3(−)* oocyst at 20 days pbm containing sporozoites. Scale bar 5 µm. **B** Top row: *orp3(−)* oocysts in midgut at 21 days pbm. Scale bar 50 µm. Middle and lower row: Mutant *orp3(−)* and wild type salivary glands, respectively, dissected from infected mosquito at 21 days pbm. Scale bar 100 µm **C** Oocyst counts from two clones of *orp3(−)* and WT infections. Oocysts were counted at 10 and 21 days pbm. Pooled data from two experiments for each strain. Ns non-significant, ****p < 0.0001, Mann-Whitney test.

The localization of ORP3 in the oocyst was investigated, using a specific, polyclonal anti-ORP3 serum (see Methods). Wild-type oocysts were probed at day 13 post blood meal (pbm), showing ORP3 localization at the periphery of the cyst (Fig. 1C). To investigate ORP3 function, we generated a *P. berghei* mutant that lacked the *orp3* gene (Supplementary Fig. 2A). Two independent *orp3(−)* clones (Supplementary Fig. 2B), constitutively expressing GFP, were analyzed for their ability to complete the life cycle. Asexual blood stage growth rates and ookinete formation were not affected, compared to wild-type (WT) parasites (Supplementary Fig. 2C). The *Anopheles gambiae* mosquito strain G3 was infected with WT and *orp3(−)* clones. At day 10 pbm, oocysts on dissected midguts were counted, revealing comparable oocyst numbers in WT and *orp3(−)* parasites. Mutant oocysts were of normal size and sporozoites were observed inside the mature oocyst (Fig. 2A). As a control, *orp3(−)* oocysts were also probed with anti-ORP3 serum, confirming the absence of ORP3 in these oocysts, even over long exposure times (Supplementary Fig. 2D). Around 21 days pbm, the majority of WT oocysts had broken and GFP-expressing sporozoites were detected in the salivary glands (Fig. 2B) while *orp3(−)* oocysts persisted in the mosquito midgut (Fig. 2B, C, and Supplementary Data 1). Both midguts and salivary glands were dissected from the same infected mosquitoes at day 21 pbm, and only mosquitoes positive for infection were considered. Only a few mutant sporozoites were detected in the salivary glands, dissected at day 21 pbm, consistent with the lack of oocyst rupture (Table 1). Furthermore, mosquitoes infected with either *orp3(−)* mutant clone failed to transmit malaria to naïve mice (Table 1).

**Table 1:** Sporozoite counts from dissected salivary glands and transmission to naive mice

	Average No. oocysts per dissected midgut	Salivary gland sporozoites/mosquito	No. dissected mosquitoes	Transmission to naive mice
WT				
EXP1	13	4000	16	2/2
EXP2	11	1700	25	2/2
*orp3(−)cl1*				
EXP1	14	5,2	19	No
EXP2	15	8,4	26	No
*orp3(−)cl2*				
EXP1	11	5,3	26	No
EXP2	9	0,1	20	No

Taken together, these findings demonstrate that ORP3 is essential for oocyst rupture and sporozoite release, mirroring the function of ORP1 and ORP2, and suggests that the three proteins may form a trimeric complex similar to NF-Y, which assembly triggers oocyst excystation.

### ORP3 is expressed at the oocyst periphery

**Fig. 3:**
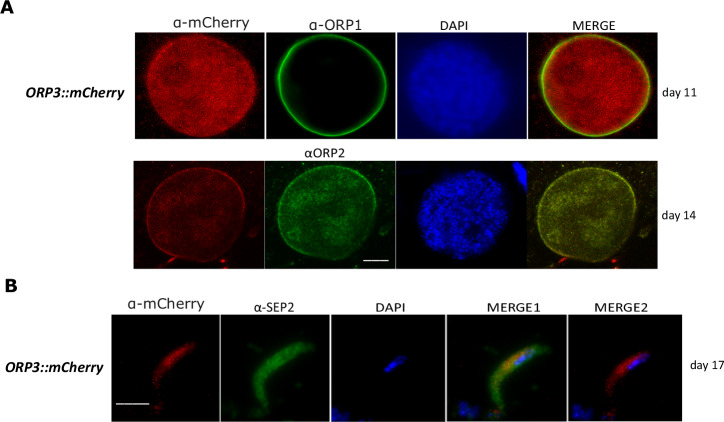
Localization of ORP3 in oocysts and sporozoites. **A** Localization of ORP3 and ORP2 at day 7 and 14 pbm. ORP2 and ORP3 co-localize at day 14 pbm, when the oocyst is ready to rupture. Scale bar, 20 µm. **B** ORP3::mCherry localization in the sporozoite. ORP3::mCherry (red) is detected in midgut and salivary gland sporozoites and localizes near the nucleus. An antibody recognizing SEP2 (green) labels sporozoite cytoplasm. Scale bar, 20 µm.

To further investigate the role of ORP3 during mosquito stages in vivo, we determined the localization of ORP3 using a transgenic line expressing endogenous ORP3, fused at the C-terminus to the mCherry protein (Supplementary Fig. 2C, E). It was previously demonstrated that ORP1 resides at the oocyst capsule^7^. To assess potential co-localization of ORP3 with ORP1 or ORP2, immunofluorescence assays were performed using antibodies that recognize peptides in ORP1 and ORP2, together with anti-mCherry antibodies to detect ORP3::mCherry (see Methods). At day 11 pbm, ORP3::mCherry mainly localizes inside the oocyst, whereas ORP1 resides at the cyst capsule (Fig. 3A). At mature stages (day 14 pbm), ORP2 and ORP3::mCherry co-localize inside the cyst and, possibly, at the periphery (Fig. 3A).

ORP3 was also detected in sporozoites, collected from mechanically ruptured oocysts, at day 17 pbm. ORP3::mCherry also partially co-localized with SEP2, a protein detected in the sporozoite cytoplasm^20^ (Fig. 3B). However, the ORP3 signal was restricted to an area near the sporozoite nucleus, whereas SEP2 was found dispersed throughout the cytoplasm.

### ORP3 interacts with the ORP1/2 heterodimer in yeast

**Fig. 4:**
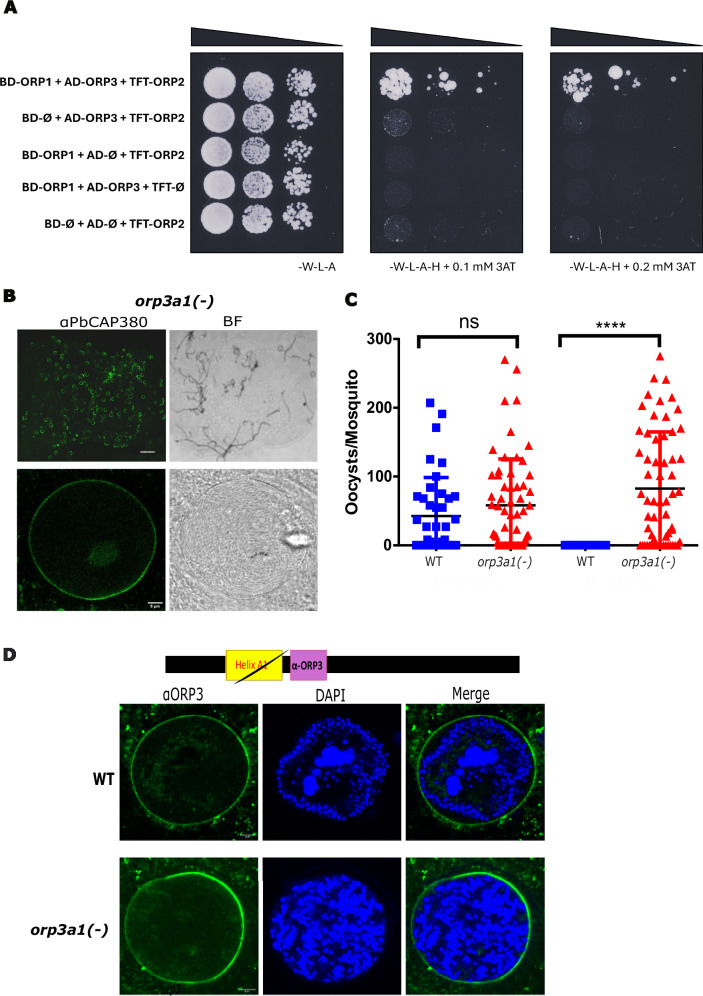
In vitro interaction between the three ORPs and role of ORP3 helix A1 during oocyst development. **A** Yeast three-hybrid assay demonstrates interaction between ORP1, ORP2, and ORP3. The ability of ORP1, ORP2, and ORP3 to form a trimeric complex was evaluated using a yeast three-hybrid assay. *S. cerevisiae* strain AH109 cells were co-transformed with combinations of pGBKT7::ORP1 (BD-ORP1), pGADT7::ORP3 (AD-ORP3), pTFT::ORP2 (ORP2), or empty vector controls. Positive clones were cultivated in SD media and cultures normalized to an OD^600nm^ of 0.5, serially diluted, and spotted onto SD media lacking tryptophan, leucine, histidine, and adenine (-W-L-H-A), supplemented with 0.1 mM 3-aminotriazole (3-AT). Growth only occurred when all three proteins were expressed simultaneously (BD-ORP1, AD-ORP3, and ORP2), indicating that ORP1 and ORP3 require ORP2 to interact. **B** Phenotypic analysis of the *orp3a1(−)* mutant during oocyst development. *Orp3a1(−)* mutant oocysts midgut collected at 21 days pbm probed with the capsule marker PbCAP380. Scale bar 200 µm. In the lower panel, oocyst enlargement showing sporozoites are fully formed inside the oocyst. Scale bar 5 µm. **C**. Oocyst counts from *orp3a1(−)* and WT infections. Oocysts were counted at 10 and 21 days pbm. Pooled data from two experiments for each strain. Ns, non-significant, ****p < 0.0001, Mann-Whitney test. **D** *Orp3a1(−)* oocyst probed with a serum recognizing an ORP3 epitope immediately after the region A1 removed in the mutant showed no difference in ORP3 localization compared to WT oocyst. Scale bar 5 µm.

The ability of ORP3 to assemble into a trimeric complex with the ORP1/2 heterodimer was evaluated using a yeast three-hybrid assay, as described in the Methods section. A 97- amino acid N-terminal construct of ORP3, encompassing helix A1 (Fig. 1A), was tested for its ability to physically interact with the HFD dimer of ORP1/2. Co-transformations of *S. cerevisiae* strain AH109 cells, with various combinations of plasmids expressing or not the three proteins, confirmed that growth on selective media (with tryptophan, leucine, adenine, and histidine) was only possible when all three proteins were co-expressed simultaneously. The yeast-three-hybrid controls also indicated that ORP1 and ORP3 do not interact and that ORP1 and ORP2 heterodimerization is required as a prerequisite for complex formation with ORP3 (Fig. 4A).

### An ORP3 mutant lacking helix A1 produces oocysts that do not rupture

To investigate the role of helix A1 in oocyst rupture, and to test its potential interaction with the ORP1/2 heterodimer, a mutant named *orp3a1(−)* was generated in which the 87 bp region, encoding for helix A1, was deleted (Supplementary Fig. 2F). Asexual blood stage growth rates, gametocytes and ookinete formation were not affected, upon comparison with WT parasites (Supplementary Fig. 2G). *Anopheles gambiae* strain G3 mosquitoes were infected with the WT and *orp3a1(−)* clone, in three independent experiments, and the resulting phenotypes were analyzed. Oocysts were normally formed, and sporozoites were detected in mature oocysts (Fig. 4A, B). However, oocysts from mutant parasites, stained using the PbCAP380 capsule marker, persisted in midguts for up to 3 weeks after the blood meal. Instead, the majority of cysts in WT-infected midguts were broken and sporozoites were released around day 14 pbm (Fig. 4C). After 21 days pbm, salivary glands from the *orp3a1(−)* mutant and WT-infected mosquitoes were dissected. Sporozoites were only detected in WT samples, while no mutant sporozoites were detected in the salivary glands, suggesting that they remain trapped within unruptured oocysts (Table 2).

**Table 2:** Sporozoite counts from dissected salivary glands and transmission to naive mice

	No. of dissected mosquitoes	Salivary gland sporozoites/mosquito	Transmission to naive mice
WT			
EXP1	20	2950	2/2
EXP2	20	4517	2/2
*orp3a1(−)*			
EXP1	20	0	No
EXP2	20	0	No
EXP3	20	0	

To determine if the *orp3a1(−)* mutant was altered in protein localization, an anti-ORP3 serum, recognizing an epitope located immediately after helix A1, was used for immunolocalization. In the mutant, ORP3 was detected at the oocyst periphery, in agreement with observations made for the WT, indicating that its localization was not altered in this mutant (Fig. 4D).

### CSP is mis-localized and prematurely processed in *orp(−)* oocysts

**Fig. 5:**
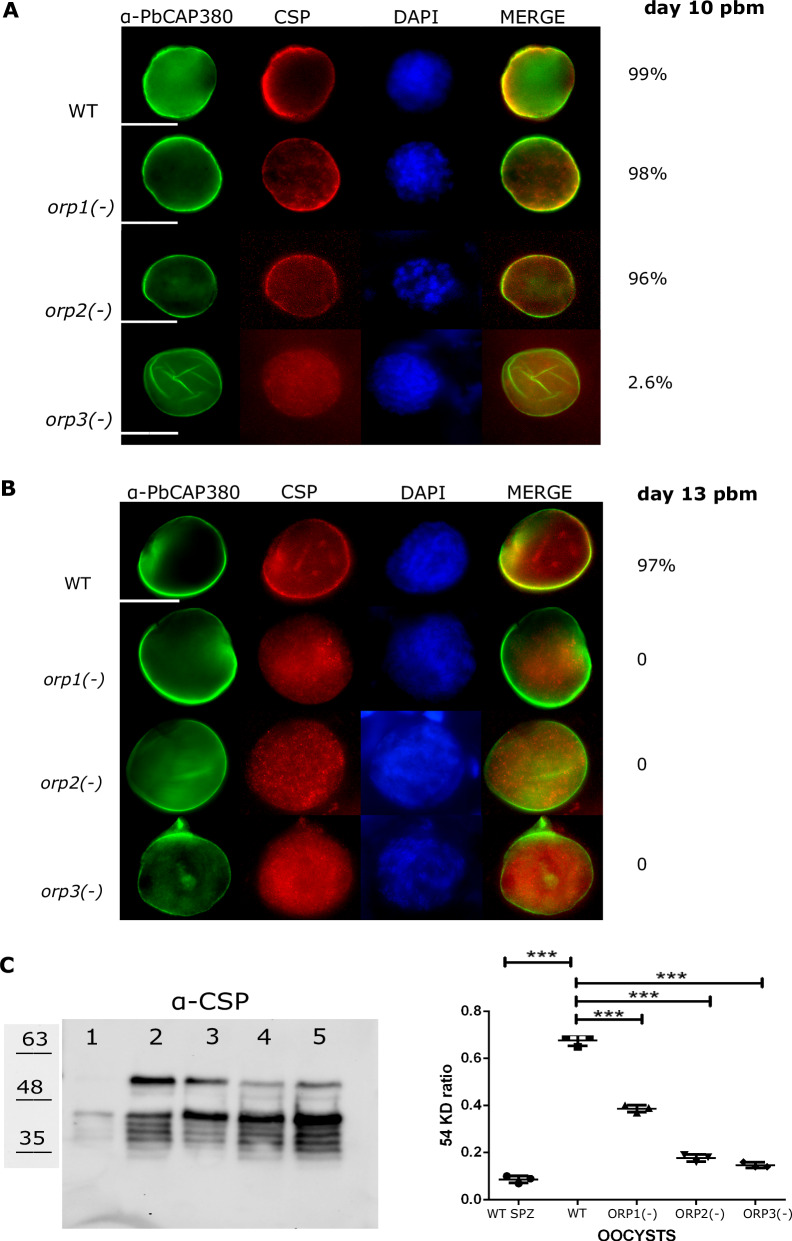
Localization of CSP in *orp(−)* mutants. **A** Oocysts at day 10 pbm. CSP (red) resides at the cyst membrane in WT, *orp1(−)* and *orp2(−)* parasites but is localized in the cytoplasm in *orp3(−)* oocysts. CAP380 (green) highlights the capsule. On the right, percentage of CSP correct localization at the membrane is indicated. **B** CSP localization in mature oocysts at day 13 pbm. CSP (red) is localized in the cytoplasm in *orp(−)* parasites. On the right, percentage of CSP correct localization at the membrane is indicated. **C***Left panel.* Western blot analysis of CSP in mutant oocysts. Lane 1 WT sporozoites from salivary glands. Lanes 2–4 oocysts from infected midguts day 14 pbm; lane 2 WT, lane 3 *orp1(−)*, lane 4 *orp2(−)*, lane 5 *orp3(−)*. *Right panel.* Estimates of the ratio of cleaved/uncleaved CSP by measuring the intensities of each band (54 kDa vs (54 + 44 kDa)). Differences are significant *p* < 0.0001, Student’s *t* test. Error bars denote SEM.

The CSP is highly expressed in WT oocysts and localizes to the plasma membrane^21,22^. At day 10 pbm, however, the majority of *orp3(−)* mutant oocysts displayed CSP mis-localization to the cytoplasm (Fig. 5A), with only a few oocysts (2.6%, of a total of 500 counted oocysts, Supplementary Data 2) retaining normal membrane localization of the protein. This led us to investigate whether CSP membrane localization was also affected in the *orp1(−)* and *orp2(−)* mutants. Indeed, membrane localization of CSP, assessed at 13 days pbm, was lost in all *orp(−)* mutant oocysts, with the protein being visualized in the cytoplasm with very low signal at the membrane, in contrast to WT oocysts, where the protein remained at the periphery (Fig. 5B).

CSP is processed during sporozoite maturation and hepatocyte infection, and forms of CSP with different molecular weight have been described during sporozoite maturation in mosquitoes^11^. To determine if mis-localization of CSP was due to abnormal processing, samples of WT and mutant parasites were analyzed by western blot (Fig. 5C, left panel). Pools of five infected midguts, collected at 14 days pbm from respectively WT, *orp1(−)*, *orp2(−)* and *orp3(−)* parasites and a WT salivary gland sporozoite sample (2000 sporozoites), were probed with an anti-CSP monoclonal antibody. CSP was detected in all samples, although the absolute quantification of the protein could not be determined due to variable oocyst numbers in each sample. However, the two major forms of CSP, with molecular weights (MWs) of 54 kDa and 44 kDa, were detected in all samples, as previously described^23,24^. Mutant oocysts exhibited an increased proportion of the processed, lower MW form relative to WT (Fig. 5C right panel). Specifically, the percentage of processed CSP in mutant oocysts from *orp1(−)* parasites was about 50% more compared to WT, and 80% more in *orp2(−)* and *orp3(−)* oocysts (Fig. 5C right panel, Supplementary Data 3). Samples from WT and mutant parasites were also probed with a serum recognizing the C-terminus of CSP^23,24^. As shown in Supplementary Fig. 3, the serum mainly recognizes the higher MW form of CSP in WT parasites while, while in the mutants, only the lower MW form of CSP was detected. This confirms that CSP is prematurely processed in the mutants. These results were reproducible across multiple independent experiments, suggesting that the ORPs have a direct or indirect role in controlling CSP processing.

### Mutant sporozoites are motile but fail to develop into exoerythrocytic forms (EEFs)

**Fig. 6:**
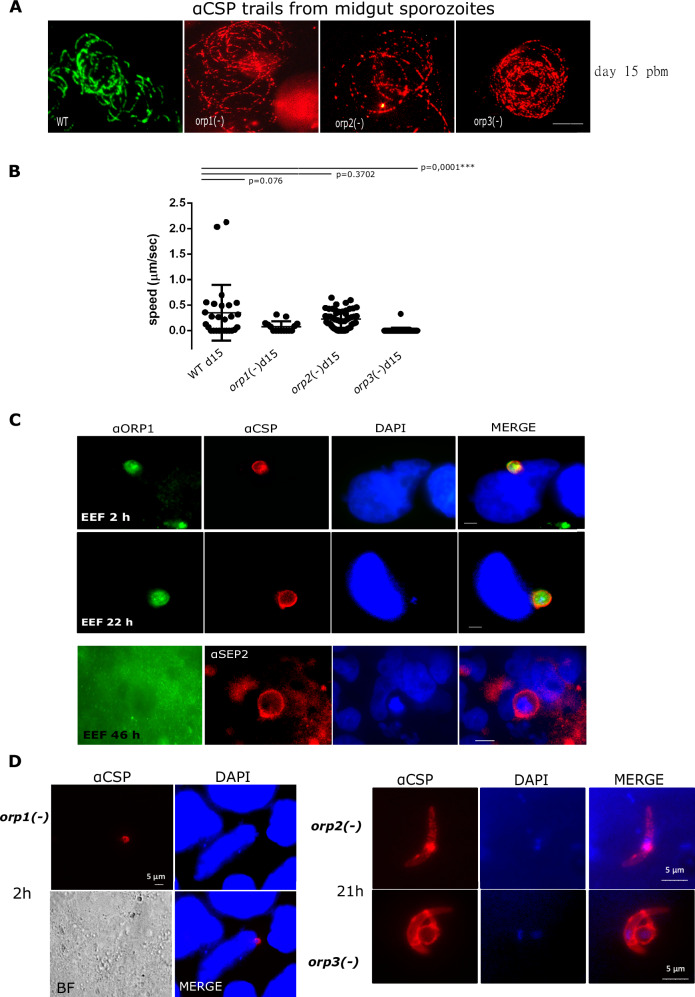
Motility of *orp(−)* mutant parasites and development into exoerythrocytic forms. **A** Gliding assay on microscope slides of WT, *orp1(−)* and *orp2(−) orp3(−)* sporozoites at 15 days pbm. CSP was deposited from all three parasites in a dotted pattern. Scale bar 50 μm. **B** Comparison of the speed of WT and the mutants lacking ORP proteins. Sporozoites were analyzed 15 days and 21 days pbm from mechanically ruptured oocysts. Statistical significance (Student's *t* test) is indicated above the histogram for each comparison. **C** ORP1 is detected in WT parasites infecting liver cells at 2 and 22 h post infection. Early EEFs are stained with CSP (red) while ORP1 is stained in green. Later EEFs development, at 46 h post infection, are marked with SEP2 antibody, a marker of the parasitophorous vacuole, and no ORP1 is detected. **D***Left panel.* Liver cells infected with *orp1(−)* sporozoites marked with anti-CSP antibodies (red) at 2 h post invasion. *Right panel.* *Orp2(−)* and *orp3(−)* mutant parasites are not able to invade and develop into EEFs at 21 h post infection of the liver cells. Parasites are stained with CSP antibodies (red), nuclei are visible in blue.

As previously shown, none of the *orp(−)* mutants were able to infect mice when mosquitoes were allowed to feed on the animals (Tables 1, 2) and^7^. To investigate if this was due to a defect in gliding motility, we assessed WT and *orp(−)* sporozoite motility in vitro using the gliding assay. This assay visualizes the trails deposited by motile sporozoites during gliding using the anti-CSP antibody for detection^25,26^. Sporozoites, mechanically released from oocysts at 16 days pbm, were allowed to glide on glass slides. The trails from all strains were comparable, and showed a dotted pattern (Fig. 6A), suggesting that mutant sporozoites have no major motility defects. To examine this in more detail, we determined the speed of individual sporozoites of the same strains. Parasites were collected from oocysts at 15 days pbm, spotted on microscope glass slides and the movements were recorded in videos. This time point was chosen as WT sporozoites are released in the hemocoel after 15–16 days pbm. The actual speed of the sporozoites was determined by manual tracking after subtraction of the motility of background particles. No significant difference in speed was found when comparing the speed of *orp1(−)* and *orp2(−)* mutants and WT sporozoites (Fig. 6B), whereas *orp3(−)* parasites showed a dramatic decrease in motility compared to WT. Furthermore, ORP expression in liver cells, and their importance in infection, was evaluated. To identify the presence of ORPs during exoerythrocytic development, Huh7 liver cells were infected with WT sporozoites. ORP1 was detected at 2 and 21 h post infection in EEFs that were recognized by their αCSP label (Fig. 6C). EEFs were stained with the SEP2 antibody to detect the parasitophourous vacuole^20^ at 46 h after infection, but no ORP1 signal was detected. In contrast, ORP2 and ORP3 proteins were not detected in EEFs at any time point.

Mutant *orp(−)1-3* sporozoites were tested for their ability to infect liver cells. Human huh7 hepatocytes were incubated with sporozoites from each *orp(−)* mutant and infection was followed up to 46 h post infection. After 2 and 21 h, EEFs were detected using αCSP, performing three replicates per time point. Around 20 fields were checked in each experiment, and three independent replicates were carried out. Very early EEFs were detected only in one experiment at 2 h post infection in *orp1(−)* infected liver cells (Fig. 6D), whereas no EEFs were detected at the later time point. In *orp2(−)* and *orp3(−)* infected liver cells, no EEFs were detected, whereas mutant sporozoites were degenerated and/or sticky when visualized 21 h post invasion (Fig. 6D), suggesting that the lack of *orp(−)* causes a defect in their ability to invade/develop into EEFs.

## Discussion

We previously showed that dimerization between the histone fold domains (HDFs) of two oocyst rupture proteins (ORP1 and ORP2) is required for *Plasmodium* oocyst rupture and infective sporozoite egress^7,14^. We take this finding a step further and show that ORP complex formation and oocyst excystation involve a third oocyst rupture protein (ORP3).

The HFDs of ORP1 and ORP2 are homologs of NF-YB and NF-YC subunits, respectively^7^. NFY is active as a biological trimer, with the third subunit (NF-YA) conferring DNA binding specificity^15,16^. In particular, NF-YA contains two α-helices: helix A1 that mediates trimerization with the NF-YB/C dimer, and helix A2 that mediates DNA binding^16^. Now we identified a third ORP (ORP3), present at the oocyst periphery, that contains a domain that is conserved with the NF-YA trimerization helix A1, suggesting that it may bind the ORP1/2 dimer. In line with the non-nuclear localization of the ORPs and thus unlikely role in DNA binding, helix A2 is absent in ORP3 (Fig. 1). It should be noted that none of the three ORPs are related to NF-Y or other CCAAT-binding proteins outside the fore-mentioned, conserved domains^7^.

The localization of the three ORPs largely overlaps in mature oocysts, with evidence suggesting that they are present in the capsule prior to excystation^7^. Functional analyses revealed that all three ORP proteins share the same phenotype; deletion mutants form oocysts and sporozoites develop but their egress from the oocyst is blocked. Furthermore, the mutant sporozoites lacking ORP1 and ORP2 display normal motility, while in *orp3(−)* motility is reduced.

Our experimental evidence shows that trimerization and oocyst rupture can be specifically attributed to the A1 helix of ORP3 as *orp3a1(−)* mutant parasites, devoid of helix A1, exhibited blocked sporozoite egress from the oocyst. Furthermore, physical interaction between helix A1 and the ORP1/2 HFD dimer was demonstrated in yeast.

The parallel between ORP proteins and NF-Y subunits is fascinating and supports the role of the ORP HFD dimer as an adapter module for the interaction with a third molecular partner, as in NF-Y. At a functional level, *Plasmodium* ORPs are unlikely to be bona fide transcription factors, considering their localization (so far reported always outside the nucleus) and their function during sporozoite egress. The ORP complex may be considered an example of moonlighting proteins in *Plasmodium*, with protein domains being re-purposed during parasite development, as described for other organisms^27^. Interestingly, ORPs represent only the second reported example of an HFD-containing protein with a function outside the nucleus, the first being the Ras-specific nucleotide exchange factor Son-of-Sevenless (Sos), which was described as a membrane binding factor^28^.

The precise molecular function of the ORP complex is difficult to envisage, as few proteins are known to play a role in oocyst excystation and most of those are expressed in the developing sporozoites rather than in the capsule per se. It has been suggested that sporozoite motility is necessary for egress^2^, but both our data as well as studies on mutants of CSP reveal that other factors also play a role. In the case of CSP, mutants expressing different protein regions fused to GFP were shown to produce motile sporozoites, but egress did not take place^29^.

Mutant sporozoites lacking ORP proteins cannot establish infections in mice (7; Tables 1 and 2), and here we also show that they do not develop into EEFs in liver cells in vitro. *Orp1(−)* mutants invade the cells, but EEFs do not develop, while the two other mutants remain outside the hepatocyte. Together, these data imply that the ORPs are involved in two independent processes: the mechanism of excystation and establishment of the infection in liver cells. The function in the sporozoite stage however, differs between the three proteins. ORP1 and ORP3 are expressed intracellularly in sporozoites, while ORP2 is not detected in this stage. The reason for the block in infectivity, at least in *orp2(−)* is therefore dependent upon some other factor, while the diminished motility of *orp3(−)* could contribute to this phenotype. ORP1, on the other hand, may play a role during the establishment of infection in hepatocytes. Further studies on the role of ORP1 in the liver stages will necessitate a conditional mutant to specifically deplete ORP1 in the sporozoite stage, while allowing oocyst rupture to take place.

CSP is a GPI-anchored protein localized at the internal side of the oocyst cell membrane and at the surface of the sporozoite. Processing of CSP is important during both during sporozoite gliding and sporozoite invasion of liver cells^24^. It is less clear whether processing is important for sporozoite egress. We found that in all three *orp(−)* mutants, CSP is mis-localized when oocysts are ready to break, and is pre-maturely processed into its lower MW form, possibly due to cleavage of the N-terminus. Whether these two phenotypes are related is difficult to assess. However, CSP undergoes conformational changes in the mosquito salivary glands, allowing correct sporozoite motility and infectivity^30^. It is possible that mis-regulated CSP processing, occurring in the midgut sporozoites of *orp(−)* mutants, rather than in salivary gland sporozoites, leads to incorrect folding of CSP, rendering the sporozoites non-infectious. In summary, our data underline the importance of ORP trimer formation for inducing oocyst burst, via an unknown mechanism (direct or indirect) that may involve CSP processing. The requirement for a physical interaction between the three ORPs highlights the possibility of designing inhibitory molecules that prevent the interaction between the ORP1/2 HFD dimer and helix A1 in ORP3, as a potential strategy to prevent malaria transmission.

## Methods

### Ethics statement

We have complied with all relevant ethical regulations for animal use and all work was carried out in full conformity with Greek regulations and laws on animal experiments. In Greece, these issues are covered by the Presidential Decree (160/91) and law (2015/92), which implement the directive 86/609/EEC from the European Union and the European Convention for the protection of vertebrate animals used for experimental and other scientific purposes and the new legislation Presidential Decree 56/2013. The experiments were carried out in a certified animal facility, and the protocol was approved by the FORTH Ethics Committee (101/14-12-2020) and by the Prefecture of Crete (license number # 106323, 29/04/2021and #184636, 29/05/2024). Mice parasitaemia was monitored after injection of *P. berghei* parasites through GIEMSA staining and humane endpoints were predefined. Animals exhibiting signs of severe sufferance were sacrificed in accordance with institutional guidelines to minimize paining. Mice were euthanised through inhalation of carbon dioxide.

Intramuscular injections of 50 mL mixture of 3.3 mg/mL xylazine and 33 mg/mL ketamine were used as anesthetic and no surgery was carried out.

### Statistics and reproducibility

For statistical analysis of Motility of *orp(−)* mutant parasites Students t-test was applied.

For the phenotypic analysis of *−* and *orp3a1(−)* mutants, *p*-values from pooled data from two experiments for each strain were calculated using the Mann–Whitney U-test.

All analyses were performed using GraphPad Prism v8.0.

### Yeast three-hybrid assay

The cDNA sequences of the histone fold domains of ORP1 (amino acid residues 768–860) and ORP2 (amino acid residues 1–111), and the N-terminus of ORP3 (amino acid residues 40–137), encompassing helix A1, were subcloned into the pGBKT7, pTFT, and pGADT7 vectors, respectively (Gene Universal Inc). *Saccharomyces cerevisiae* strain AH109 (Takara Bio Inc.) cells were co-transformed with plasmids expressing the ORP proteins and the corresponding empty control vectors using the Gietz yeast transformation protocol^31^. Transformants harboring pGBKT7, pGADT7, and pTFT vectors (with and or without inserts) were selected on Synthetic Drop-out (SD) media depleted of tryptophan, leucine, and adenine (–W –L –A).

To assess the ability of ORP1, ORP2 and ORP3 to form a trimeric complex, positive clones were cultured in SD –W –L –A liquid media, the optical density at 600 nm wavelength (OD^600nm^) was adjusted to 0,5, serially diluted and spotted onto SD–W–L–A (control) and SD depleted of tryptophan, leucine, adenine and histidine (–W–L–H–A) with 0,1 mM 3-aminotriazole (3-AT, Sigma A8056 Sigma-Aldrich). Plates were incubated at 28 °C for 4 days, and the interaction was visually assessed by checking the growth of yeast cultures spotted on selective media.

### Parasites, mice and mosquito maintenance

*P. berghei* ANKA 8417HP strain was used throughout the study and was maintained in Theiler’s Original mice or CD1 mice (Harlan Sprague Dawley, Inc.). Mice were male or female and ∼6–8 weeks when infected. *A. gambiae* strain G3 mosquitoes were reared as described^32^. Mosquito infections were carried out by offering *A. gambiae* strain G3 to mice infected with WT and mutant. The mutant strains *orp1(−)* and *orp2(−)* have been previously described^7^.

### Generation of mutant parasites

The pBAT plasmid^33^ was used to generate the final constructs to create *orp3(−)* and *orp3::mCherry* mutants.

The transgenic parasite line expressing ORP3::mCherry was generated by introducing the coding region of mCherry in the endogenous locus by double crossover. To this end, the last 966 bp of the orp3 coding region (amplified with primers ORP3CODfor and ORP3cod2rev; Table S1), were fused in frame with the gene encoding mCherry in the pBAT vector using the enzymes *SacII* and *SpeI*. A 554 bp fragment of the 3′ UTR of *orp3* (amplified with primers ORP3tg-3′ for and ORP3tg-3′ rev) was inserted via *SphI* and *SacI* restriction sites. The last step is the insertion of a 636 bp homologous region for recombination just after the orp3 3′UTR region amplified by ORP3tg-omfor and ORP3tg-omrev (Table S1) in the *ApaI–AatII* sites. Finally, the plasmid was linearized using *AatII* and *SacII* enzymes before transfection.

The *orp3(−)* parasites were generated by introducing the drug-selectable cassette linked to a high expression GFP cassette in pBAT by double crossover. For *orp3(−)*, the gene was replaced via a double crossover, targeting the left side of the gene with a 880 bp PCR fragment (primers YAS1 and YAS2) inserted using the enzymes *SacII* and *SpeI* and the right side with a PCR fragment of 516 bp (primers YAD1 and YAD2) in the *ApaI* and *XhoI* restriction sites. The plasmid also encodes the recyclable hDHFR-yFcu cassette, which encodes anti-folate resistance, and the gene for GFP regulated by the PbHSP70 promoter. After transfection, GFP-fluorescent red blood cells were sorted and intravenously injected into two CD1 mice and kept under pyrimethamine selection. When parasitaemia was established, parasites were sorted again. Parasites were diluted to obtain the injection of one single cell per mouse and once parasitemia was established samples were genotyped by PCR. The two clones were derived from independent transfections. All transfections were performed using the Human T Cell Nucleofector Kit (Lonza, cat. no. VPA-1002).

The *orp3a1(−)* mutant was generated using CRISPR/Cas9 methodology, as previously described^34^. Briefly, the homologous recombination (HR) template was delivered as a linear fragment, while the ribozyme-gRNA-ribozyme (RGR) cassette was cloned into a plasmid encoding the sgRNA sequence, flanked by Hammerhead and Hepatitis Delta Virus ribozymes, which self-cleave to release functional sgRNA^35^. The HR template was assembled by nested PCR from three overlapping fragments.

Fragment 1 (960 bp) was amplified with primers Fr1_Fw and Rev_pam, fragment 2 (114 bp) with Fw_pam and Fr2_Rev, and fragment 3 (846 bp) with Fr3_Fw and Fr3_Rev. Primers were designed to provide a 32 bp overlap between fragment 1 and fragment 2, and a 13 bp overlap between fragment 2 and fragment 3, ensuring efficient recombination. The Helix A1 deletion was introduced via primer Fr2_Rev: of its 35 nucleotides, the 5′-proximal 22 nt annealed immediately upstream of Helix A1, while the 3′-terminal 13 nt annealed 13 bp downstream of the targeted region. PCR amplifications were performed with Phusion High-Fidelity DNA Polymerase (NEB) under conditions recommended by the manufacturer.

For assembly, fragment 1 and fragment 2 were fused by overlap extension PCR, followed by nested PCR with primers Fr1_Fw and Fr2_Rev to generate fragment 4. Fragment 2 and fragment 3 were fused in the same manner, with amplification by primers Fr2_Fw and Fr3_Rev to yield fragment 5. Fragments 4 and 5 were subsequently joined by overlap extension and nested PCR using primers Fw_nested and Rev_nested, producing the complete HR template.

For overlap extension reactions, purified PCR fragments (Qiagen PCR Purification Kit) were combined at equimolar ratios (300 ng total DNA per 50 μL reaction) and amplified without primers under the following cycling conditions: 15 cycles of 98 °C for 10 s, 40 °C for 30 s, and 62 °C for 2.5 min. Nested PCR was performed using 5 μL overlap extension product as template in a 100 μL reaction with the respective primer pairs, using the following program: 98 °C for 10 s, 53.6 °C for 30 s, and 62 °C for 45 s.

The RGR sequence was synthesized by GenScript (https://www.genscript.com/) in the pET17 vector. The RGR fragment was excised and subcloned into the pSL1433 vector^35^ using *EcoRI* and *NheI* restriction sites. Correct insertion and orientation were confirmed by PCR with two primer pairs: Act2flag-RGR-Fw1 (annealing within the Hammerhead ribozyme of the RGR cassette) with pSL1433_Rev_cloning (annealing within the vector backbone), and pSL_Fw_cloning (vector-specific) with Act2flag-RGR_Rev1 (annealing within the HDV ribozyme).

Transfected parasites were intravenously injected into OlaTo mice and subjected to pyrimethamine selection for five consecutive days.

Following drug withdrawal, parasites rapidly lost the episomal plasmid carrying the sgRNA in the absence of selective pressure. This window was exploited to enrich for transformants by FACS, based on GFP expression encoded within the pSL1433 vector. GFP-positive parasites were sorted, reinjected intravenously into mice, and clonal transgenic lines were derived by limiting dilution.

Genotyping of wild-type and mutant clones was performed using four primer combinations: Fw-genotyping/Rev-nested, Fw-genotyping/Seq3rev, Fw_pam/Rev-genotyping, and Fw-nested/Rev-genotyping. The wild-type allele produced amplicons of 776 bp, 709 bp, 244 bp, and 157 bp, respectively. In contrast, the mutant allele yielded no products with the first two primer sets, while the latter two generated fragments of 990 bp and 157 bp.

All primer sequences (BIOSUPPLIES) are available in Table S1.

### Antibodies

An ORP3 polyclonal serum, recognizing the amino acid sequence KNSQNNAITTNSRNC, was purchased (Genscript) and tested in immunofluorescence assays at a 1:100 dilution.

Antibodies recognizing ORP2 were raised (Eurogentec) against a peptide present in the histone fold domain (amino acids 11–25) and were used in immunofluorescence assays at a 1:100 dilution. The monoclonal antibody 3D11, recognizing the repeat region of CSP (299), was used at a 1:6000 dilution in Western blot assay and a 1:600 dilution in IFA, and SEP2 1:400 in IFA^20^. The sera recognizing the C-terminus and the disordered region of CSP were used at a 1:5000 dilution.

#### Immunofluorescence analysis

Midguts were dissected at different days after the blood meal and fixed for 1 h in 4%*(v/v)* paraformaldehyde/1%*(v/v)* saponin in PBS. All incubations were carried out for 1 h at room temperature. After three washes in PBS/1%*(v/v)* saponin (PBSS), parasites were blocked with 5%*(v/v)* normal goat serum (NGS)/PBSS, incubated with the primary antibody diluted in PBS, washed several times in PBS after which the secondary antibodies were added (1:800 dilution). Cell nuclei were labeled with 4,6-diamidino-2-phenylindole. Sporozoites were fixed as above and permeabilized with 0.1% *(v/v)* Triton X-100 in PBS, followed by incubation with the primary antibodies, as fore-mentioned. The specificity of the immune sera was checked in parallel using pre-immune sera and controls were included to exclude nonspecific binding of the secondary antibodies. Samples were viewed using a Zeiss Axioskop 2 plus microscope and, in some cases, analyzed using a Zeiss LSM 510 confocal laser scanning microscope. Images were further processed using ImageJ.

### Western blotting

For western blotting, crude extracts from dissected midguts or sporozoites were analyzed by SDS-PAGE using a 12% polyacrylamide gel and the proteins were transferred to a Protran 0.22 μm membrane (Whatman). Primary and horseradish peroxidase-conjugated secondary antibodies were diluted in PBS-Tween (0.05%*(v/v)*) before addition to the blots. The blots were developed using the ECL system (SuperSignalWest Pico, Thermo Scientific), according to the manufacturer’s instructions.

Estimates of the ratio of cleaved/uncleaved CSP were determined by measuring intensities of each band on the blot using an ImageJ plugin BandpeakQuantification, using the lane-profile method^36^. Three blots were analyzed, and tests for statistical significance were carried out using Student’s *t* test in pairwise comparison mutant versus WT.

### Motility assay

Sporozoite motility on a glass surface was performed, as previously described^25^. Briefly, midguts from infected mosquitoes were dissected and the sporozoites released by gentle pressure. The gliding assay using CSP trails detection was performed according to ref. ^26^. Sporozoite motility was recorded using an optical OLYMPUS IX-70 microscope with CCD at one frame per second for over one minute. Videos were analyzed with manual tracking using ICY to determine the speed of individual sporozoites, expressed as μm/sec. The displacement was calculated from the position of sporozoites in all frames; actual sporozoite speed was determined after subtraction of the displacement of background particles. About 150 sporozoites were analyzed per timepoint.

### Liver cell invasion

Cells of the Huh7 human hepatoma cell line were cultured in RPMI medium supplemented with 10% fetal calf serum, 1% non-essential amino acids, 1% penicillin-streptomycin, 1% glutamine, and 10 mM HEPES (pH 7) (all from Gibco/Invitrogen) and were maintained at 37 °C under 5% CO_2_. For infection, *P. berghei* sporozoites obtained from freshly dissected mosquito midguts were added to cell monolayers seeded 24 h earlier on coverslips in complete medium and used when confluence was ∼80–90%. Cell culture plates were centrifuged for 5 min at 1.800 *× g* to promote contact of the parasites with the Huh7 cell monolayer.

### Statement for all methods

This is to confirm that all methods were carried out in accordance with relevant guidelines and regulations.

### Statement for the reporting of animal experiments ARRIVE guidelines

This is to confirm that all methods are reported in accordance with ARRIVE guidelines and the check list is provided

### Reporting summary

Further information on research design is available in the Nature Portfolio Reporting Summary linked to this article.

## Supplementary information

Supplementary Information

Description of Additional Supplementary Files

Supplementary data 1

Supplementary data 2

Supplementary data 3

Reporting Summary

Transparent Peer Review file

## Data Availability

All data are available as “Supplementary Data files”. All other data are available from the corresponding author on reasonable request. All uncropped western blot images can be found in the Supplementary Information file.
