# Supplementary material for: Sporozoite egress from Plasmodium oocysts requires a trimeric NF-Y–like complex
Source: Commun Biol. 2026 Apr 27;9:886. doi: 10.1038/s42003-026-10147-6 (PMC13323700; doi:10.1038/s42003-026-10147-6)
Supplement: Supplementary file 1 — Supplementary Information [file 42003_2026_10147_MOESM1_ESM.pdf]

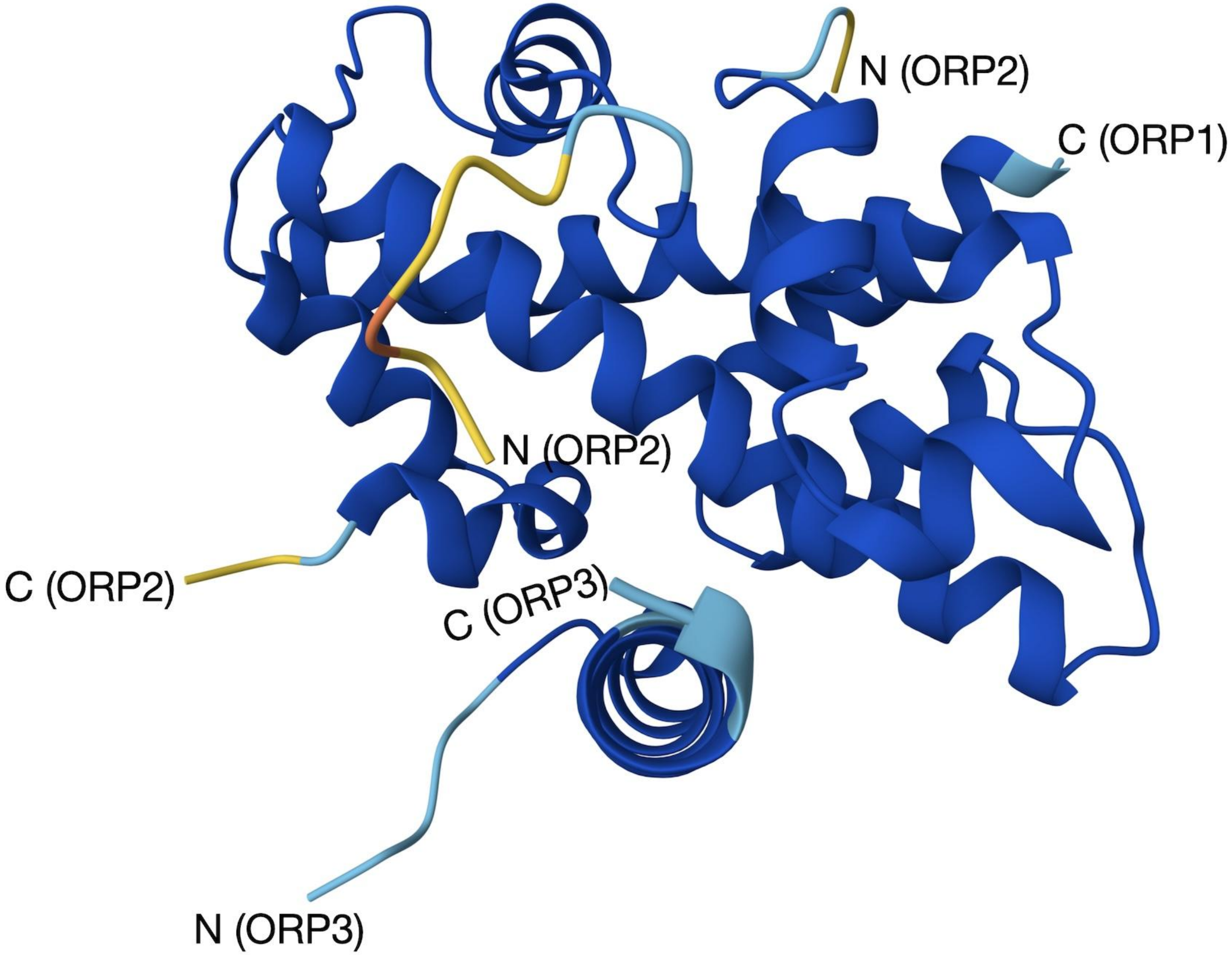

Supplemental figure 1. The structural organization of the ORP trimer. Complex formation between the HFD dimer formed between ORP1 (residues 774 to 860) and ORP2 (residues 5 to 111), with residues 40 to 72 of ORP3 that house the region containing conserved helix A1, was predicted using AlphaFold (1). The N- and C- termini of each ORP is indicated. Ribbon color coding is in line with AlphaFold confidence scores with very low confidence regions in orange (plDDT < 50), low confidence regions in yellow (plDDT > 50), high confidence regions in turquoise (plDDT > 70) and very high confidence regions in blue (plDDT > 90) (17). This figure was generated using ChimeraX version 1.9 (2).

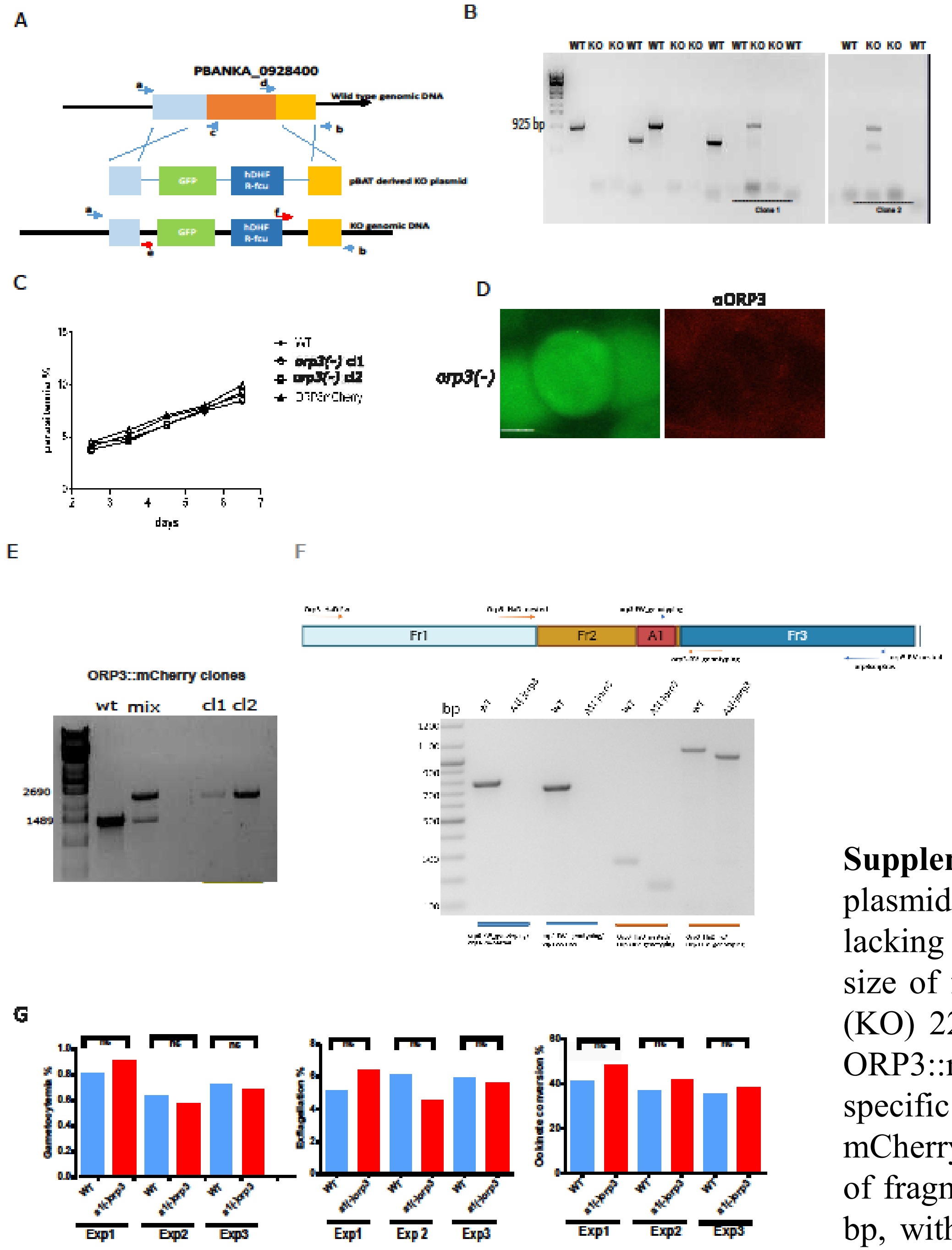

Supplemental figure 2. Generation of mutant parasites. A. Schematic representation of the plasmid used for generation of *orp3(-)* parasites. **B.** Generation of the two mutant parasites clones lacking *orp3*. PCR genotyping showing the presence of the mutant band in the clones. Expected size of fragments: primers Sext/Sint (WT) 985 bp, Sext/mCherryback (KO) 1100bp, YAD2/hA (KO) 2200 bp, Dext/Dint (WT) 605 bp. **C.** Growth rate of asexual stages of the *orp3(-)* and ORP3::mCherry mutants compared to WT **D.** *orp3(-)* oocysts expressing GFP probed with ORP3 specific serum. **E.** Generation of two clones of mutant parasites encoding ORP3 fused to mCherry. PCR genotyping showing the presence of the mutant band in the clones. Expected size of fragment for the mutant using primers codfor/mcherryback is 2166 bp while for WT is 1462 bp, with the primers codfor/D2YA. **F.** Generation of the *orp3a1(-)* mutant. **G.** Growth rate of asexual and sexual stages of the *orp3a1(-)* mutant compared to WT.

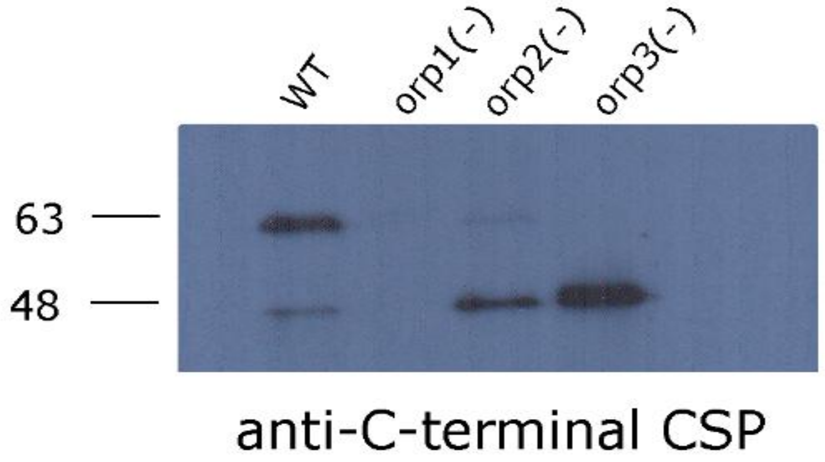

Supplemental Figure 3. Midgut oocysts samples from WT and mutant parasites were probed with two different sera recognizing the C-terminus of CSP

Supplemental references

1. Ballabio, F., Bertaso, C., Villa, M., Livero, O., Del Cont Bernard, A., Russo, R., Gessmann, R., et al. Targeting the Histone-fold-domain dimerization interface of Oocyst Rupture proteins from *Plasmodium berghei* for anti-Malarial therapies. *FEBS J.* doi: 10.1111/febs.70389. Epub ahead of print (2026).

2. Pettersen, E.F., Goddard, T.D., Huang, C.C., Meng, E.C., Couch, G.S., Croll, T.I., Morris, J.H. & Ferrin T.E. *Protein Sci.* **30**, 70-82 (2021).

full size blots orp3KO genotyping
(Suppl Figure 2)

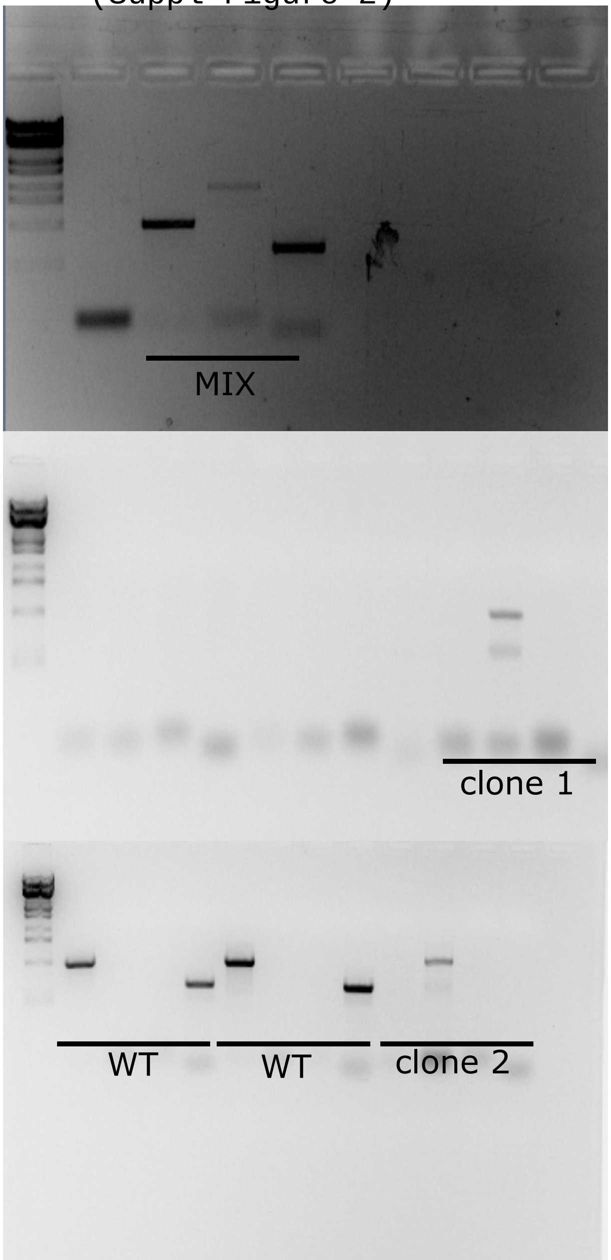

full size blot ORP3mCherry genotyping
(Suppl figure 2)

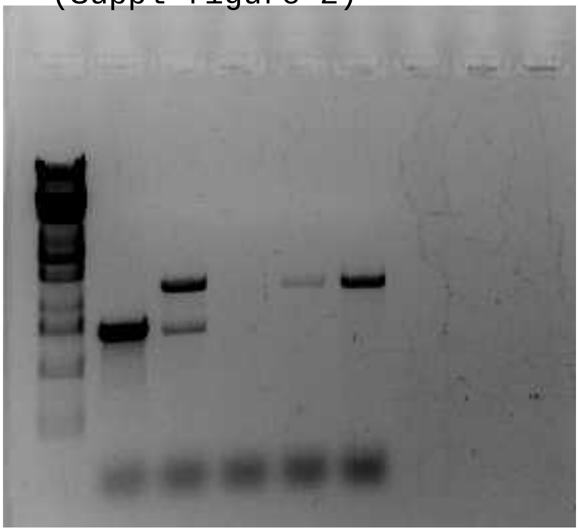

full size anti CSP immunoblot
(Fig 5 C)

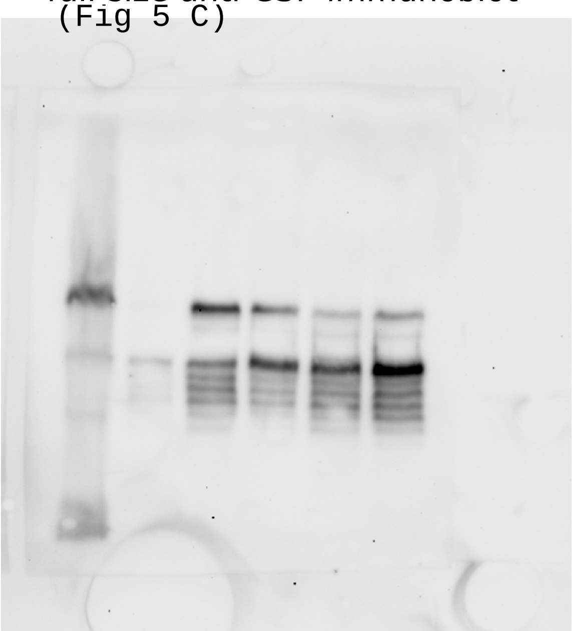

Uncropped supplementary Figure 3

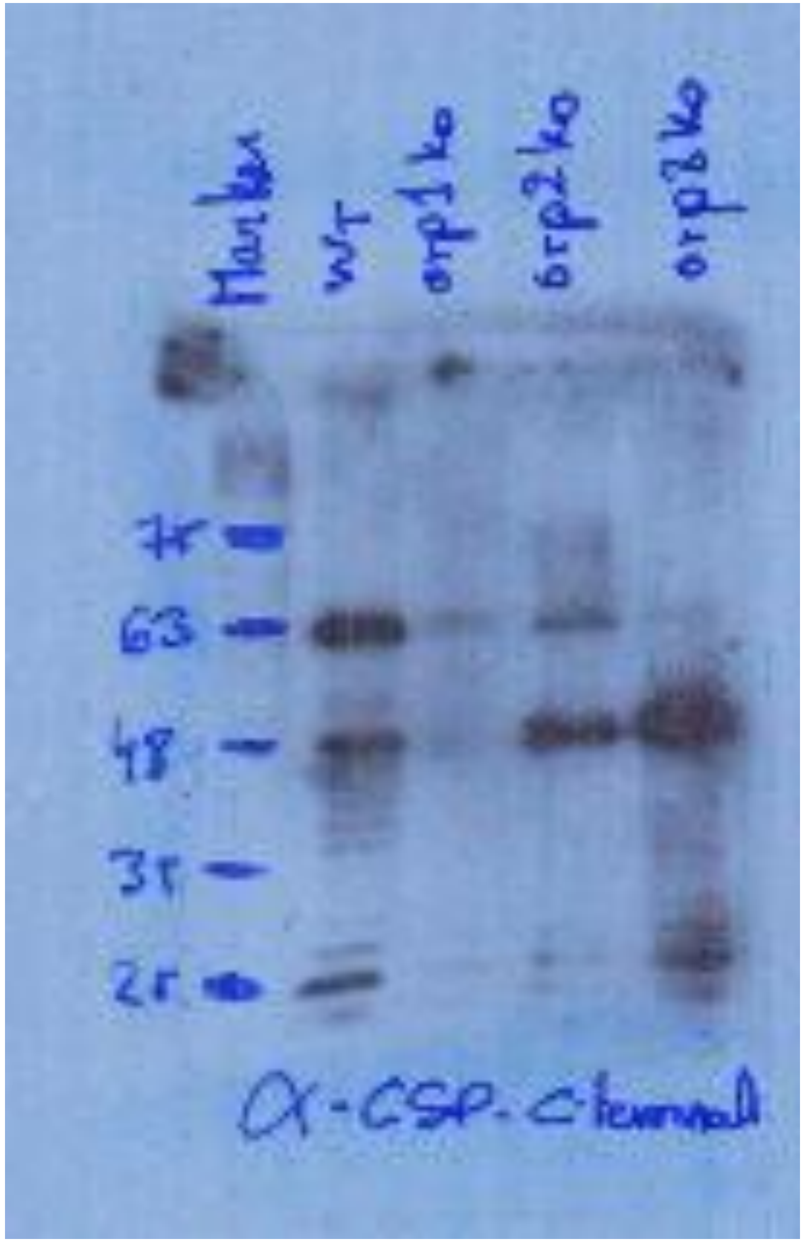

Table S1. Sequences of primers used in generation and genotyping of mutants.

S1	gaccCCGCGGTTTACCAGAACGGGGAG
S2	gaccACTAGTCATTCATTTCGAGGTTGG
D1	gaccGGGCCCCCTTATTCCCCCTATGACG
D2	gaccCTCGAGACGTGTTTCTTTGCTGTA
SExt	CGGTTTCAAGCCAAATG
SInt	ATATTGTTGATTTCGAGGG
DxInt	CTGTTCCTTTGAATAGAAG
DxExt	GGCCTATGATTGTGTT
mCherry-back:	ATGATGGCCATGTTATCCTC
5'PbDHFR-back:	GCATGGGGAAAGAGTGGCTT
ORP3CODfor	gaccCCGCGGGGTAGATGGGATGGTG
ORP3CODrev:	gaccACTAGTTTCGTCATAGGGGGAATAAGGG
ORP3tg-3'for:	gaccGCATGCGTTTATTTTTCATTTCACTTA
ORP3tg-3'rev:	gaccGAGCTCAAGGCCTATGATTGTG
ORP3-omfor:	gaccGGGCCCTTCCCAATCAGCATTT
ORP3-omrev:	gaccGACGTCTGTACCATCTTAATTGA
orp3_HaD_fr1_Fw	CGGTTTCAAGCCAAATGC
orp3-HaD_fr1_RV_PAM	TCATTTCGAGGTTAGAATTCATGTTTAATGAT
orp3-HaD_fr2_Fw_PAM	ATCATTAAACATGAATTCCAACCTCGAAATGA
orp3-HaD_fr2_RV	CATAGACACTGATACCATCCCATCTACCATAATTG
orp3-HaD_fr3_Fw	A**ATCAGTGTCTATG**TTAATATTCAA
orpr-HaD_fr3_RV	AGGGTAACTTCTATTCAAAGG
orp3_HaD_Fw_nested	ATTTACCAGAACGGGGAG
Orp3-HaD_RV_nested	GTTAATACCCACATTTTGTCC
Orp3-HaD_Fw_genotyping	AAGACGAATGAATAAAATAAAGCA
Orp3-HaD_Rv_genotyping	TTGAATATTAACATAGACACTGAT
orp3seq3rev	GTTCGAAAGGTTTGGTACGC
pSL_Fw_cloning	GGCGGCCGCGCATATTA
Act2flag-RGR-Fw1	CCGTGAGGACGAAACGAGTA
Act2flag-RGR_Rev1	CGAAGCATGTTGCCCAGC
pSL1433_Rev_cloning	ACGGATAATGACCGGTAGGC
