# Supplementary material for: Sporozoite egress from Plasmodium oocysts requires a trimeric NF-Y–like complex
Source: Commun Biol. 2026 Apr 27;9:886. doi: 10.1038/s42003-026-10147-6 (PMC13323700; doi:10.1038/s42003-026-10147-6)
Supplement: Supplementary file 2 — Description of Additional Supplementary Files [file 42003_2026_10147_MOESM2_ESM.pdf]

Description of Additional Supplementary Files

File name: Supplementary Data 1

Description: The source data behind the graphs in the paper

File name: Supplementary Data 2

Description: ORP3KO clone BC feeding

File name: Supplementary Data 3

Description: CSP measurments from ImageJ
