# Supplementary material for: Sporozoite egress from Plasmodium oocysts requires a trimeric NF-Y–like complex
Source: Commun Biol. 2026 Apr 27;9:886. doi: 10.1038/s42003-026-10147-6 (PMC13323700; doi:10.1038/s42003-026-10147-6)
Supplement: Supplementary file 7 — Transparent Peer Review file [file 42003_2026_10147_MOESM7_ESM.pdf]

nature portfolio

# Peer Review File

# Sporozoite Egress from Plasmodium Oocysts Requires a Trimeric NF-Y–Like Complex

Corresponding Author: Dr Chiara Curra

**This file contains all reviewer reports in order by version, followed by all author rebuttals in order by version.**

Version 0:

Reviewer comments:

Reviewer #1

(Remarks to the Author)
Previous work from the authors' laboratory described two proteins (ORP1 and 2), participants of a trimer required for the release of sporozoites from oocysts. In this manuscript, the authors describe the trimer's third protein (ORP3).

This work complements well previous work and provides some additional important information, such as the relationship of the trimer with the processing and function of the sporozoite coat protein CSP. Overall, experiments are carefully conducted, and the conclusions are well supported.

Major comment.
The experiments illustrated in Fig. 6D are not reliable and need to be redone. One problem is that no positive control with wild type sporozoites is shown. Another problem is that late EEFs were probed with an anti-CSP antibody. This is the wrong choice, as late EEFs do not express CSP.

Minor comments.
- The title of the Fig. 1 legend does not relate to the data shown.

- The evidence in Fig. 3A that ORP3 localizes to the periphery on day 14 is weak.

- Insert "day 10 pbm" and "day 21 pbm" under Fig. 4C.

- Line 219: change Fig. 6A to Fig. 5A.

- Lines 235 and 237: "Fig. 5C right panel" is described as "Fig. 5D" in the legend.

Reviewer #2

(Remarks to the Author)
In this manuscript, Preira and co-workers identify and investigate a third 'oocyst rupture protein' (ORP3) that appears related to the eukaryotic NF-Y complex. They show that the protein is localized at/near the oocyst capsule in late oocysts and is necessary for sporozoite egress and sporozoite infectivity to the liver. They also demonstrate interaction of ORP3 with ORPs 1 and 2 in yeast. This is very much an extension of a previous study from 2016 by the same group (Siden-Kiamos et al, Nat. Comm.) in which ORPs 1 and 2 were shown to do the same thing, so the novelty factor is limited.

Specific comments:

General: I take some issue with the use of capsule 'rupture' when referring to sporozoite egress. To me the term 'rupture' suggests a catastrophic event that causes mass release of sporozoites, but this is not supported by most of the literature that describe a more gentle sporozoite egress through a small opening. I am not expecting the ORPs to be renamed, but the process overall could be better worded.
Abstract: Line 43: 'Extra-Erythrocytic' should be 'exoerythrocytic'.

Introduction
Line 65-66: The protein TRP1 is also associated with the capsule and necessary for sporozoite egress (Klug et al 2017, eLife) so this study should be mentioned and cited here.
Line 69: The authors should also mention a study by Saeed et al 2023 (Sci Rep) which shows sporozoite egress via small holes using live fluorescence microscopy.
Lines 89-90: PDB 9RIF is not accessible, nor is reference 16, so this information is currently not usable.
Lines 91-99: these are a direct repeat of lines 82-90.

Results
Line 112, 114. It is not clear that we are dealing with paralogues, so better refer to as homologues.
Line 119: ..alphafold predicts most of the protein ORP3 to be intrinsically disordered.. This observation likely reflects the fact that there are very few amino acid sequences in the databases with homology to ORP3 (apart from its small region with the alpha helix) and consequently the rest comes out as 'spaghetti'. The confidence if the prediction is too low and you cannot know if it is unstructured without resolving the structure experimentally.
Line 167: ..as for ORP1.. The authors must mean ORP2, because ORP1 is at the oocyst periphery by day 7 according to their own work.
Line 179: Should you talk of 'in vitro' interaction when using Y3H? Better to state interaction in yeast. On this point, the authors should test for in vivo interactions between ORPs in oocysts using immunoprecipitation-type experiments, since they have ORP-specific antibodies and fluorescent protein-fused ORP parasite lines.
Line 219: Fig. 6A should be 5A
Line 222: ..alteration in the localization of CSP in the membrane of ORP1- and ORP2- mutants was observed (Figure 5A).. I do not see a difference and the legend even says there is no difference. Maybe they mean 5B?
Lines 242-245: lots of typos and poor grammar, please rewrite.
Line 246: EEF is exoerythrocytic forms
Line 248-249: This describes work already published and the wrong citation is given (it's not reference 3). Regarding motility, the authors should investigate if the sporozoites of the ORP-KO lines display motility inside the oocysts as shown for the ECP1 knockout (Aly & Matuschewski) or do they only become motile after release as shown for the TRP1 knockout (Klug & Frischknecht)? This is a simple experiment that would tell you much more about the potential relationship between the ORPs and TRP1.
Lines 263-269 and fig 6CD: The data should be quantified.

Discussion
Line 272: you cannot start a Discussion with 'These three proteins…'
Line 313: 'normal and motile sporozoites' is misleading, as ORP3- sporozoites appear to have significantly reduced speed (Fig. 6). And what does that potentially mean for sporozoite infectivity to the liver? Please elaborate.
Generally, the discussion is a bit superficial and in my view the most interesting question is not addressed, i.e . what does it mean from an evolutionary perspective to find a homologue of NF-YA that seems to interact with homologues of NF-YB and NF-YC? To me this suggests that the ORPs have an orthologous relationship with NF-YA, B and C and have been acquired as a 'set' with only the 'interacting' domains staying conserved and the rest of the protein chains changing to carry out their new function. This topic can and should be better explored.

Version 1:

Reviewer comments:

Reviewer #1

(Remarks to the Author)
The authors positively addressed my suggestions.

In their rebuttal, they state that "Liver cells were also infected with orp(-) mutants and the infection was followed up to 46 hours post infection. Only in one experiment a EEF at very early stage was detected for orp1(-)".

I strongly suggest that they incorporate into the manuscript the fact only in one experiment a EEF at very early stage was detected for orp1(-).

Reviewer #2

(Remarks to the Author)
The authors have made appropriate modifications.

**Open Access** This Peer Review File is licensed under a Creative Commons Attribution 4.0 International License, which permits use, sharing, adaptation, distribution and reproduction in any medium or format, as long as you give appropriate credit to the original author(s) and the source, provide a link to the Creative Commons license, and indicate if changes were made.
In cases where reviewers are anonymous, credit should be given to 'Anonymous Referee' and the source.
The images or other third party material in this Peer Review File are included in the article's Creative Commons license, unless indicated otherwise in a credit line to the material. If material is not included in the article's Creative Commons license and your intended use is not permitted by statutory regulation or exceeds the permitted use, you will need to obtain permission directly from the copyright holder.
To view a copy of this license, visit https://creativecommons.org/licenses/by/4.0/

**Rebuttal letter**

**We thank you very much both reviewers for their comments; here we respond point-by-point to the issues raised on our paper. We strongly believe that we increased the quality of the paper.**

Reviewer #1 (Remarks to the Author):

Previous work from the authors' laboratory described two proteins (ORP1 and 2), participants of a trimer required for the release of sporozoites from oocysts. In this manuscript, the authors describe the trimer's third protein (ORP3).

This work complements well previous work and provides some additional important information, such as the relationship of the trimer with the processing and function of the sporozoite coat protein CSP. Overall, experiments are carefully conducted, and the conclusions are well supported.

Major comment.
The experiments illustrated in Fig. 6D are not reliable and need to be redone. One problem is that no positive control with wild type sporozoites is shown. Another problem is that late EEFs were probed with an anti-CSP antibody. This is the wrong choice, as late EEFs do not express CSP.

**REPLY: Experiments with WT parasites were done at 2, 21 and 46 hours post infection using different markers to detect early (CSP) or late (SEP2) exoerythrocytic forms; ORP1-3 specific sera and appropriate markers have been used in IFA. We now included figure 6C showing that ORP1 is expressed in early EEFs, revealed by CSP co-localization experiments, but not in later forms, identified by SEP2 co-localization experiments. ORP2 and ORP3 are not detected in EEFs.**

**Liver cells were also infected with *orp(-)* mutants and the infection was followed up to 46 hours post infection. Only in one experiment a EEF at very early stage was detected for *orp1(-)*.**

**Minor comments.**
- The title of the Fig. 1 legend does not relate to the data shown.

**REPLY: The title has been corrected**

- The evidence in Fig. 3A that ORP3 localizes to the periphery on day 14 is weak.

**REPLY: ORP3 localization at cyst periphery is suggested in figure 1C and figure 4D. In figure 3 we used an ORP3::mCherry strain to make double immunofluorescence assay using an mCherry antibody with ORP2 and ORP3, tentatively assessing a possible co-localization between ORP2.**

**We wrote again part of the paragraph**

- Insert “day 10 pbm” and “day 21 pbm” under Fig. 4C.

**REPLY: It is now inserted**

- Line 219: change Fig. 6A to Fig. 5A.
**REPLY: we corrected the name**

- Lines 235 and 237: “Fig. 5C right panel” is described as “Fig. 5D” in the legend.

**REPLY: This is now corrected**

Reviewer #2 (Remarks to the Author):

In this manuscript, Preira and co-workers identify and investigate a third ‘oocyst rupture protein’ (ORP3) that appears related to the eukaryotic NF-Y complex. They show that the protein is localized at/near the oocyst capsule in late oocysts and is necessary for sporozoite egress and sporozoite infectivity to the liver. They also demonstrate interaction of ORP3 with ORPs 1 and 2 in yeast. This is very much an extension of a previous study from 2016 by the same group (Siden-Kiamos et al, Nat. Comm.) in which ORPs 1 and 2 were shown to do the same thing, so the novelty factor is limited.

Specific comments:

General: I take some issue with the use of capsule ‘rupture’ when referring to sporozoite egress. To me the term ‘rupture’ suggests a catastrophic event that causes mass release of sporozoites, but this is not supported by most of the literature that describe a more gentle sporozoite egress through a small opening. I am not expecting the ORPs to be renamed, but the process overall could be better worded.

**REPLY: About “rupture”, a valid comment. The paper by Klug and Frischknecht describing slow exit of the sporozoites (2017) was published after our Nat. Comm paper (2016) so perhaps unfortunate about use of terms but we modified in the text.**

Abstract: Line 43: ‘Extra-Erythrocytic’ should be ’exoerythrocytic’.
**REPLY: We corrected the term**

Introduction

Line 65-66: The protein TRP1 is also associated with the capsule and necessary for sporozoite egress (Klug et al 2017, eLife) so this study should be mentioned and cited here.

**REPLY: we now mentioned the paper in the suggested paragraph.**

Line 69: The authors should also mention a study by Saeed et al 2023 (Sci Rep) which shows sporozoite egress via small holes using live fluorescence microscopy.

**REPLY: we now mentioned the paper in the suggested paragraph.**

Lines 89-90: PDB 9RIF is not accessible, nor is reference 16, so this information is currently not usable.

**REPLY: Reference 16 (17) has now been published (it was accepted at the time of submission) and the PDB, which was on hold for publication, has now been released**

Lines 91-99: these are a direct repeat of lines 82-90.
**We deleted the repeated paragraph**

Results

Line 112, 114. It is not clear that we are dealing with paralogues, so better refer to as homologues.

**REPLY: We now changed the term in the text.**

Line 119: ..alphafold predicts most of the protein ORP3 to be intrinsically disordered.. This observation likely reflects the fact that there are very few amino acid sequences in the databases with homology to ORP3 (apart from its small region with the alpha helix) and consequently the rest comes out as ‘spaghetti’. The confidence if the prediction is too low and you cannot know if it is unstructured without resolving the structure experimentally.

**Reply: Alphafold does not generate its 3D predictions based solely on sequence identity to proteins of known structure. It is not a homology modelling program rather it also relies on deep learning approaches to infer interactions between residues. Low Alphafold confidence scores can indirectly indicate potential intrinsically disordered protein (IDP) regions. Since IDPs lack a defined structure, they cannot be reliably predicted and, therefore IDP regions consistently show low Alphafold confidence scores.**

**We believe that the AlphaFold prediction of ORP3 as an IDP is highly reliable, as it is fully consistent with sequence-based IDP predictions for the ORP3 sequence. IDPs can now be accurately predicted from sequence alone based on characteristic features such as the depletion or absence of nonpolar residues (that typically form a hydrophobic core in globular proteins) and enrichment in polar residues. IDP predictors such as IDPred use knowledge of sequences of amino acid regions that are not visible in electron density in PDB structures, suggesting them to belong to dynamic, unstructured regions. Even without using an IDP predictor, simple inspection of the ORP3 sequence (see below) makes it is immediately evident that it is likely to be intrinsically disordered. In fact, ORP3 contains a high number of asparagines (27.5% of the total amino acid content) spread throughout the sequence except for in the A1 helix (purple).**

**MNSNLEMNGTLEINNINNVINPNCIFSNFQNETNNYGRWDGVIYVNEKQYDRILKRRMNKIKQE
MEKKKKISVYVNIQKKKNSQNNAITTNSRNSGGIENINKINNINNVANTAKIVNSNIHNNNMNRFL
TNNEHYNNGNNTIYGFDINNNDSKYNSNQYNKNNDNNYAQFYENIKYASYMKNSKNNDKKN
NGHNVNICNISNGNNQTNGYNNQINNNDTDNTYLNNYNIMYNSNINPSDDYKKYMNMYDIN
ARNTVNNIELTDGQIVNNYNETYHKIGNVSKYNSVPNLSNIGSIPNVVTLNNVEIGQNVGINRNT
NQMDKLIIMKNNQNMMHVDNINNFNNFSVPLNRSYPYSPYDE**

**It is highly improbable that a structural biologist would endeavour to solve the structure of a protein containing such extensive unstructured regions using experimental methods. Furthermore, we have not been able to produce the full-length protein either in bacterial cells or in the LEXSY expression system. We have however, successfully produced the construct (residues 40-137) used in the Y3H studies in recombinant form in E. coli. Circular dichroism analyses of the purified protein show that it is indeed unstructured. Collectively, these observations provide strong evidence that ORP3 is indeed highly unstructured. In any case, the main structural element relevant to this work is the A1 helix that is predicted structured with high Alphafold confidence.**

Line 167: ..as for ORP1.. The authors must mean ORP2, because ORP1 is at the oocyst periphery by day 7 according to their own work.

**REPLY: the paragraph has been modified and the mistake was corrected**

Line 179: Should you talk of 'in vitro' interaction when using Y3H? Better to state interaction in yeast. On this point, the authors should test for in vivo interactions between ORPs in oocysts using immunoprecipitation-type experiments, since they have ORP-specific antibodies and fluorescent protein-fused ORP parasite lines.

**REPLY: We modified "*in vitro*" to "in yeast"**

**Unfortunately, our attempts to pull down either ORPs using specific antibodies failed. In immunofluorescence assay the signal is clear and the respective controls for which we used the mutant parasites are negative to the specific ORP serum, but we get cross reaction in western blot analysis when using infected midguts. A preliminary attempt using ORP2::mcherry transgenic line failed because there was not enough material**

**perhaps because the protein is difficult to extract from the oocyst on the midgut, something which is supported by the negative results from mass spectrometry of oocyst extracts (Preira et al. 2024). It is therefore not easy to set up immunoprecipitation experiments with the reagents we have, although we agree with the reviewer that such experiments would be important for understanding the role of the ORPs.**

Line 219: Fig. 6A should be 5A

**REPLY: we corrected it**

Line 222: ..alteration in the localization of CSP in the membrane of ORP1- and ORP2- mutants was observed (Figure 5A).. I do not see a difference and the legend even says there is no difference. Maybe they mean 5B?

**REPLY: We meant 5B, corrected**

Lines 242-245: lots of typos and poor grammar, please rewrite.

**REPLY: We rewrote the sentence.**

Line 246: EEF is exoerythrocytic forms

**REPLY: we corrected the name**

Line 248-249: This describes work already published and the wrong citation is given (it's not reference 3). Regarding motility, the authors should investigate if the sporozoites of the ORP-KO lines display motility inside the oocysts as shown for the ECP1 knockout (Aly & Matuschewski) or do they only become motile after release as shown for the TRP1 knockout (Klug & Frischknecht)? This is a simple experiment that would tell you much more about the potential relationship between the ORPs and TRP1.

**REPLY: We reported the reference of the previous work. *orp(-)* mutant motility is indeed an interesting point. We never saw motility inside the oocysts in video imaging (up to 2 hours). We included the sentence.**

**"For this reason, we checked by gliding assay the ability of mutant sporozoites to glide and speed."**

Lines 263-269 and fig 6CD: The data should be quantified.

**REPLY: we added a sentence provide the number of fields counted (20 per experiments) and the number of EEFs visualized (only one at 2 h post infection in orp1(-) experiments)**

Discussion

Line 272: you cannot start a Discussion with 'These three proteins...'

**REPLY: we modified the words**

Line 313: 'normal and motile sporozoites' is misleading, as ORP3- sporozoites appear to have significantly reduced speed (Fig. 6). And what does that potentially mean for sporozoite infectivity to the liver? Please elaborate.

**REPLY: we removed this statement**

Generally, the discussion is a bit superficial and in my view the most interesting question is not addressed, i.e . what does it mean from an evolutionary perspective to find a homologue of NF-YA that seems to interact with homologues of NF-YB and NF-YC? To me this suggests that the ORPs have an orthologous relationship with NF-YA, B and C and have been acquired as a 'set' with only the 'interacting' domains staying conserved and the rest of the protein chains changing to carry out their new function. This topic can and should be better explored.

**REPLY: We thank the reviewer for this comment. Indeed it is an interesting finding, though we don't have the expertise in protein evolution that would be necessary to analyse properly this aspect. However, the discussion has been re-elaborated and several sections have been amplified.**

**Commented [ISK1]:** I don't think we can say something intelligent but we can insert the sentence along this lines suggested.

**Rebuttal letter**

**We really thank the reviewers for their important comments; here we respond point-by-point to the reviewer 1 issue.**

REVIEWERS' COMMENTS:

Reviewer #1 (Remarks to the Author):

The authors positively addressed my suggestions.

In their rebuttal, they state that “Liver cells were also infected with orp(-) mutants and the infection was followed up to 46 hours post infection. Only in one experiment a EEF at very early stage was detected for orp1(-)”.

I strongly suggest that they incorporate into the manuscript the fact only in one experiment a EEF at very early stage was detected for orp1(-).

**R: we have modified the text (lanes 253-256)**

Reviewer #2 (Remarks to the Author):

The authors have made appropriate modifications.
